# RNA Methylation by the MIS Complex Regulates a Cell Fate Decision in Yeast

**DOI:** 10.1371/journal.pgen.1002732

**Published:** 2012-06-07

**Authors:** Sudeep D. Agarwala, Hannah G. Blitzblau, Andreas Hochwagen, Gerald R. Fink

**Affiliations:** 1 Whitehead Institute, Cambridge, Massachusetts, United States of America; 2 Department of Biology, Massachusetts Institute of Technology, Cambridge, Massachusetts, United States of America; National Cancer Institute, United States of America

## Abstract

For the yeast *Saccharomyces cerevisiae*, nutrient limitation is a key developmental signal causing diploid cells to switch from yeast-form budding to either foraging pseudohyphal (PH) growth or meiosis and sporulation. Prolonged starvation leads to lineage restriction, such that cells exiting meiotic prophase are committed to complete sporulation even if nutrients are restored. Here, we have identified an earlier commitment point in the starvation program. After this point, cells, returned to nutrient-rich medium, entered a form of synchronous PH development that was morphologically and genetically indistinguishable from starvation-induced PH growth. We show that lineage restriction during this time was, in part, dependent on the mRNA methyltransferase activity of Ime4, which played separable roles in meiotic induction and suppression of the PH program. Normal levels of meiotic mRNA methylation required the catalytic domain of Ime4, as well as two meiotic proteins, Mum2 and Slz1, which interacted and co-immunoprecipitated with Ime4. This MIS complex (Mum2, Ime4, and Slz1) functioned in both starvation pathways. Together, our results support the notion that the yeast starvation response is an extended process that progressively restricts cell fate and reveal a broad role of post-transcriptional RNA methylation in these decisions.

## Author Summary

Cellular differentiation involves the limitation of cellular potential in response to developmental cues. Budding yeast cells differentiate in response to nutrient availability. In the presence of nutrients, cells divide mitotically by producing round, yeast-form buds. Under nutrient limitation, cells can either divide under a pseudo-hyphal (PH) foraging program or undergo meiosis to form protective spores. We show here that developmental commitment occurs in two distinct phases. When nutrients were removed, cells first became committed to a starvation response, during which they entered the meiotic program. If nutrient limitation persisted, cells became committed to meiosis and sporulation. By contrast, if nutrients were returned at this point, cells synchronously initiated PH foraging growth. We found that both sporulation and PH growth were governed by RNA methylation, and we identified an mRNA–methyltransferase complex comprising Mum2, Ime4, and Slz1 as a central regulator of these developmental trajectories. Our results indicate that the yeast starvation response is an extended developmental process and reveal a fundamental role for post-transcriptional RNA modification in controlling cell fate.

## Introduction

Upon nutrient limitation, diploid cells of the yeast *Saccharomyces cerevisiae* can undergo two distinct developmental responses. In the presence of a fermentable carbon source (*e.g.*, dextrose or galactose), starvation induces a modified mitotic division that produces elongated daughter progeny termed pseudohyphae (PH) [1]. Reiterated cell division under this PH program forms chains of elongated cells on solid agar, allowing yeast to forage for nutrients [2], [3]. In contrast, cells engage in meiotic development and sporulation if starved for nitrogen in the presence of a non-fermentable carbon source (*e.g.*, acetate or glycerol). Under this program, the diploid genome is duplicated (2C to 4C) and then segregated into four haploid (1C) meiotic products encased in a spore wall. This spore structure protects haploid progeny until favorable nutrient conditions are available.

Recent findings suggest that the two developmental responses to nitrogen deprivation, PH development and meiotic sporulation, are not entirely separate pathways. First, cells that are returned to mitotic growth from meiotic prophase produce elongated buds, reminiscent of PH cells [4]. Second, PH development and sporulation share a set of regulatory factors. Genes that are necessary for meiotic induction, *IME1* and *IME2* (Inducer of Meiosis 1 and 2, respectively) [5], [6], [7], [8], are also necessary for PH development and the subsequent formation of filaments on solid agar [9]. Furthermore, strains lacking the function of the early meiotic gene *IME4* display both meiotic defects and an increased ability to adhere to agar, a phenotype associated with PH development [10], [11].

In yeast, *IME4* encodes the sole functional member of a class of RNA-modifying enzymes conserved throughout eukaryotes [12]. These enzymes, identified by homology to the *N6*-adenosyl methyltransferase in humans, MT-A70, catalyze the post-transcriptional methylation of adenosine (to form *N6*-methyladenosine—m^6^A) in RNA. The function of this modification on mRNA is as yet unclear. *In vitro* work suggests that m^6^A enhances the translational activity of modified messages [13], whereas *in vivo* experiments suggest that this modification may play an additional role in message stability and processing [14], [15], [16]. Although this form of RNA methylation is barely detectable in yeast undergoing mitotic growth, m^6^A accumulates on mRNA molecules during meiosis [17], [18]. Strains encoding catalytically inactive alleles of *IME4* do not accumulate m^6^A and display defects in meiotic entry [18]. Ime4 modifies the transcripts of *IME1* and *IME2* under these conditions, which may explain these defects upon nutrient starvation [17].

Here, we investigated the role of mRNA methylation in the programmed response to nutrient starvation. Because earlier work had shown that cells remain capable of resuming mitotic growth until the exit from meiotic G2 (reviewed in [19]), we examined the early starvation response in temporal detail. We found that cells were already committed to a starvation response after meiotic cell cycle entry, because they formed PH buds when returned to nutrient-rich conditions. Lineage restriction during this period was partially dependent on the RNA methylation activity of Ime4, which had separable roles in promoting meiosis and inhibiting PH growth. Both functions also required Mum2 and Slz1, two poorly understood meiotic proteins that interacted with Ime4 and, like Ime4, were necessary for maintaining normal levels of meiotic mRNA methylation. These results point to a central role of mRNA methylation in coordinating starvation-induced developmental decisions in yeast.

## Results

### Return to mitotic growth from pre-meiotic S or G2 results in PH development

**Figure 1:**
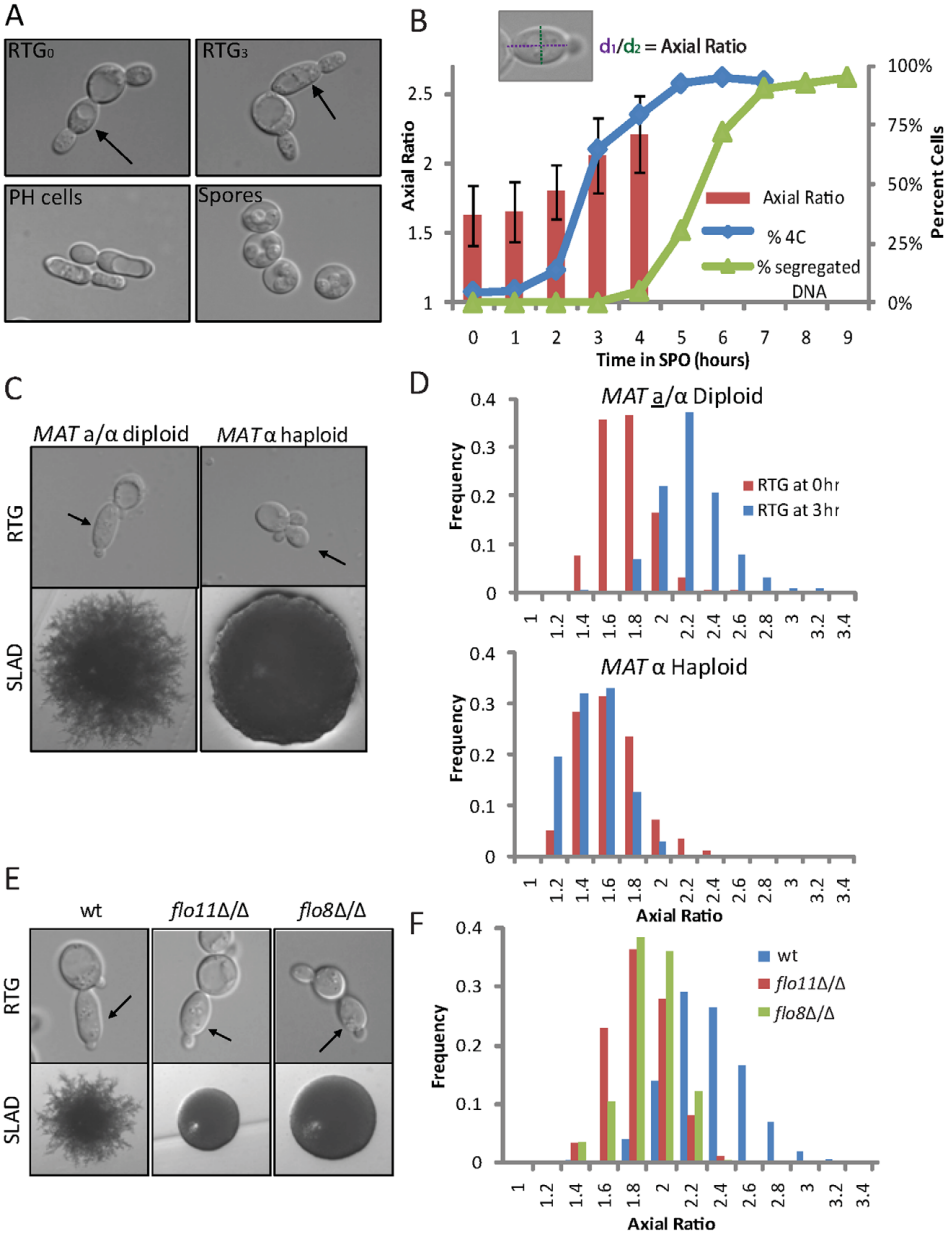
RTG from starvation results in three distinct cell morphologies. (A) Representative morphologies of daughter cells following RTG from SPO throughout a meiotic time course. Top left panel: RTG at 0 hours, *i.e.*, after growth in BYTA, (RTG_0_); top right panel: RTG at 3 hours (RTG_3_), which is comparable to PH cells from solid nitrogen medium (bottom left panel). Bottom right panel: RTG at 6 hours (RTG_6_). RTG_0_ and RTG_3_ cells were photographed after one complete cell cycle, corresponding to 160 minutes and 220 minutes after shift to rich medium, respectively (see Figure S1A). RTG_6_ cells were photographed three hours after shift to rich medium, at which point the majority of cells (>95%) had formed spores. Arrows indicate primary daughter cells upon RTG_0_ and RTG_3_. B) Quantification of axial ratio in wild-type (SAy821) cells upon RTG throughout a meiotic time course (red bars, left axis; n = 200 cells/time point) relative to percent of cells undergoing pre-meiotic DNA synthesis (*i.e.*, 4C cells) (blue diamonds, right axis, quantified by FACS, 3×10^4^ cells/time point) and percent cells undergoing meiotic divisions as assayed by DAPI staining (green triangles, right axis). Schematic at top defines axial ratio. The majority (>80%) of RTG_5_, RTG_6_, RTG_7_, and RTG_9_ cells either formed spores or remained unbudded three hours after shift to rich medium; axial ratio was therefore not quantified for these time points. C) *MAT* **a**/α diploids (SAy821) (top left panel) or *MAT* α haploids (H224) (top right panel) were returned to growth in rich medium after meiotic induction. Arrows indicate primary buds. Bottom panels represent colony morphologies after growth on SLAD for 6 days. D) Distribution of axial ratios of primary daughter cells upon RTG_3_ for strains in (C): wild-type diploid (SAy821 top panel), haploid *MAT* α (H224 bottom panel) (n = 200 cells/strain). RTG_0_ is represented in red bars, RTG_3_ in blue bars. E) Wild-type (SAy821), *flo11*Δ/Δ (SAy789) and *flo8*Δ/Δ (SAy905) daughter cell morphologies upon RTG_3_ (top panels). Arrows indicate primary daughter cells. The same strains were photographed after growth on SLAD for 6 days (bottom panels). Axial ratios are quantified in (F) (n = 200 cells/strain).

We investigated the relationship between meiotic induction and PH growth in SK1, a strain that efficiently undergoes both the PH and meiotic developmental programs [9], [20]. To analyze the early starvation response in a controlled and synchronous manner, we employed a Return-To-Growth (RTG) assay [19], [21], in which diploid cells were incubated for a given amount of time in extremely nutrient-poor liquid medium (SPO) before being returned to rich medium (YPD). Previous studies used this approach to show that cells exiting from meiotic G2/prophase are committed to meiosis and will complete sporulation even in rich medium [22], [23], suggesting that analysis of cell morphology after RTG provides a measure for the developmental potential of nutrient-starved cells. By analyzing morphological changes at hourly intervals in an RTG time course, we confirmed that cells become committed to meiosis as they exit from meiotic G2/prophase and enter the meiotic divisions. This commitment occurred 5 to 7 hours after inoculation in SPO in our strains (Figure 1A, 1B).

Analysis of cell morphology from the RTG experiment also revealed an earlier decision point, when cells became committed to a starvation response. Cells returned to growth after short starvation (0–1 hours) formed the ovoid buds characteristic of vegetative growth. However, cells that had largely completed pre-meiotic S phase or were in meiotic G2/prophase (3–4 hours after shift to SPO) were unable to do so. Instead, these cells formed elongated daughter cells that resembled PH cells (Figure 1A, 1B). Similar elongated buds are also apparent in images of RTG cells published recently by Dayani and colleagues [4]. Further analysis revealed that formation of these elongated buds paralleled PH development on solid nitrogen starvation medium (SLAD) in all aspects tested. Specifically, RTG_3_ bud elongation occurred only in diploid cells and was dependent on *FLO11*, which encodes a cell surface protein necessary for PH development, and its transcriptional regulator, *FLO8* (Figure 1C–1F) [24], [25], [26]. Furthermore, buds formed after DNA replication and mother and daughter pairs re-budded synchronously after cytokinesis (Figure S1) [3]. We termed this process RTG-PH development and conclude that, after meiotic entry, yeast cells are restricted to either meiosis or PH development. This finding suggests that nutrient-deprived yeast pass a decision point that commits them to starvation-induced differentiation.

### RTG-PH development requires *IME1* and *IME2*, but not pre-meiotic S phase

Because this developmental restriction coincided with pre-meiotic S phase (Figure 1B), we investigated whether pre-meiotic DNA replication was required for RTG-PH development. Pre-meiotic DNA synthesis in meiosis was prevented either chemically, using hydoxyurea, or genetically, by deleting the S phase cyclins *CLB5* and *CLB6* [27]. Neither condition inhibited PH development during RTG or on solid medium (Figure S2). In fact, when pre-meiotic DNA synthesis was prevented, cells instead replicated the genome upon RTG (Figure S2). These observations indicate that pre-meiotic S phase is not itself necessary for RTG-PH development. Notably, RTG-PH developmental potential followed the “readiness-for-sporulation" period as defined by Simchen as colleagues, which occurred earlier in meiosis (1 hour after induction into SPO) under our sporulation conditions (Figure S2).

**Figure 2:**
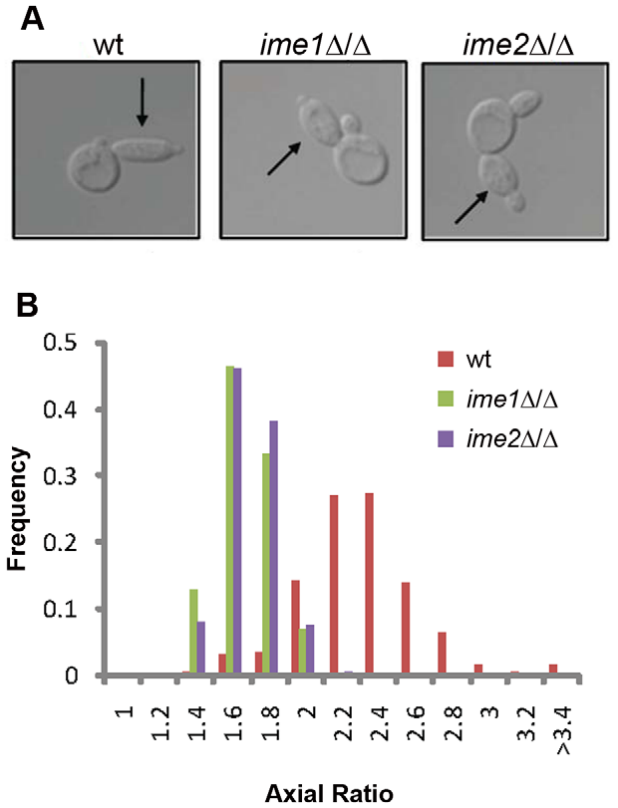
*IME1* and *IME2* are necessary for RTG PH development. A) Representative bud morphology after RTG_3_ in wild-type (SAy821), *ime1*Δ/Δ (SAy834) and *ime2*Δ/Δ (SAy859). Arrows indicate primary buds. The same strains were photographed after growth on SLAD for 6 days (bottom panels). B) Axial ratio quantification of RTG_3_ cells for strains in (A) (n = 200 cells/strain).

To probe the genetic underpinnings of RTG-PH development, we investigated the roles of factors known to regulate both meiosis and PH growth. The transcription factor Ime1 is essential for entry into the meiotic program [5]. Similarly, loss of the meiosis-specific CDK-like kinase Ime2 leads to an extreme delay in meiotic entry [28]. Both factors are also required for PH development on SLAD medium [5], [6], [7]. We found that in the absence of either *IME1* or *IME2*, cells failed to enter RTG-PH development and instead formed ovoid buds upon RTG_3_ (Figure 2A, 2B), although it should be noted that *ime2*Δ/Δ cells were able to form elongated RTG-PH buds after extended periods in SPO (Figure S3). These data suggest that, upon severe starvation, Ime1 and Ime2 promote both RTG-PH development and meiosis.

### Separable roles for *IME4* in controlling meiosis and PH development

**Figure 3:**
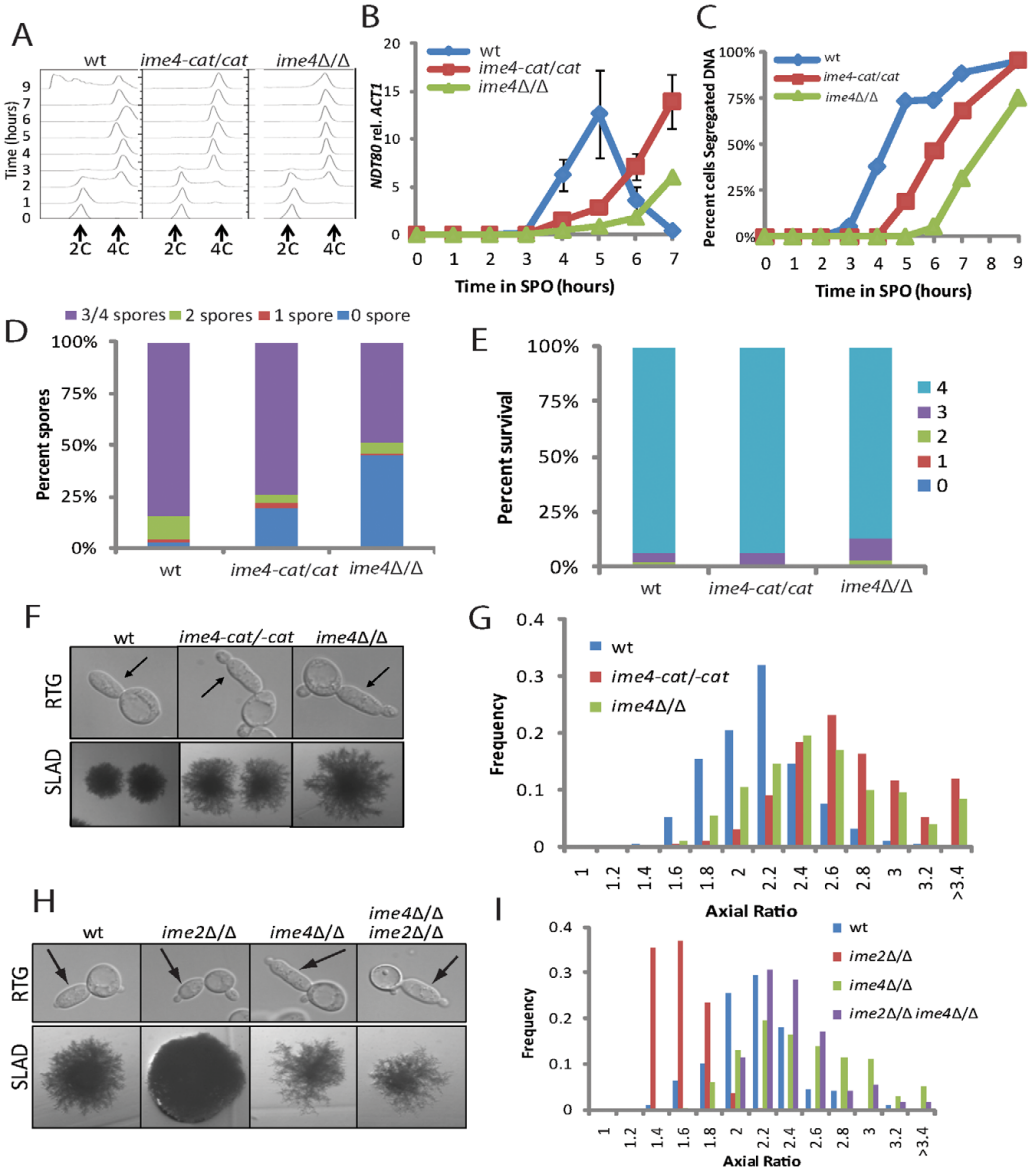
Cells encoding *IME4* mutant alleles show developmental defects. A) FACS analysis of DNA synthesis in wild-type (SAy821), *ime4-cat/cat* (SAy1086) and *ime4*Δ/Δ (SAy771) strains throughout a meiotic time course (n = 3×10^4^ cells/strain/time point). DNA content of diploid cells before DNA replication (2C) and after DNA replication (4C) is indicated. B) *NDT80* transcript levels in the strains from (A) during a meiotic time course. Transcript levels were determined by RT-PCR and normalized to *ACT1* transcript levels. C) Kinetics for meiotic nuclear divisions as assayed by DAPI DNA staining in the strains from (A) (n = 200 cells/strain). D) Number of asci with one, two, three, four, or no spores in the strains from (A) after 24 hours in SPO medium (n = 200 cell/strain). E) Spore viability in the strains from (A); legend indicates number of surviving spores upon dissection (n = 187 tetrads/strain). F) Representative images of cells from strains in (A) after RTG_3_ (top panels—arrows indicate primary buds) and colonies grown on SLAD for 6 days (bottom panels). G) Quantification of axial ratios of RTG_3_ cells shown in (F), (n = 200 cells/strain). H) Representative images of cells after RTG_3_ (top panels—arrows indicate primary buds) and colonies grown on SLAD for 6 days (bottom panels) of wild-type (SAy821), *ime2*Δ/Δ (SAy859), *ime4*Δ/Δ (SAy771) or *ime2*Δ/Δ *ime4*Δ/Δ (SAy1123) strains. Arrows indicate primary daughter cells. I) Quantification of axial ratios of RTG_3_ cells shown in (H), (n = 200 cells/strain).

Because *IME1* and *IME2* are both regulated by the RNA methyltransferase Ime4, we also analyzed meiosis and RTG-PH development in *ime4*Δ/Δ mutants. Previous work demonstrated that *IME4* promotes efficient entry into meiosis, although the severity of the meiotic entry defect of *ime4*Δ/Δ mutants varies considerably between strain backgrounds [17], [18]. In SK1, *ime4*Δ/Δ mutants exhibited only a minor delay in the initiation of meiotic DNA replication (Figure 3A), but were severely delayed in exit from meiotic G2/prophase as determined by monitoring the induction of the middle-meiotic transcription factor *NDT80* and the kinetics of meiotic DNA segregation (Figure 3B, 3C). Additionally, spore formation was substantially reduced in *ime4*Δ/Δ cells (Figure 3D). However, the viability of those spores that formed was comparable to that of wild-type cells, indicating that, although the meiotic divisions were delayed, chromosome segregation was not affected in these strains (Figure 3E). A strain carrying point mutations in the evolutionarily-conserved catalytic motif IV of Ime4 (*ime4-D349A, W351A*—referred as *ime4-cat*) [12], [18] recapitulated these phenotypes, albeit with reduced severity (Figure 3A–3E), indicating that the RNA methyltransferase activity of Ime4 contributes to the efficient progression through meiosis.

Unlike *IME1* or *IME2*, loss of *IME4* leads to increased agar adhesion [10], a phenotype associated with increased PH development. Moreover, deletion of *IME4* or mutation of its RNA methyltransferase domain resulted in bud hyper-elongation upon RTG_3_ and the formation of hyper-filamentous colonies on SLAD medium (Figure 3F, 3G). These phenotypes represented genuine forms of PH development because they were dependent on *FLO11* and *FLO8* (Figure S4). *ime4*Δ/Δ cells lacking *FLO11* or *FLO8* failed to form elongated daughter cells upon RTG and were deficient for PH colony development on nitrogen starvation SLAD medium. Thus, *IME4* acts as an inhibitor of PH development.

To test whether inhibition of PH development occurred independently of the role Ime4 plays in regulating *IME1* and *IME2*, we analyzed double mutants. As shown in Figure 3H and 3I, *ime4*Δ/Δ *ime2*Δ/Δ double mutants were hyper-elongated in both the RTG and SLAD contexts, even when compared to a time point at which *ime2*Δ/Δ cells are capable of forming RTG-PH cells (Figure S2). The hyper-elongation of *ime4*Δ/Δ cells even in the absence of *IME2* suggests that *IME4* regulates PH growth in part independently or downstream of *IME2*-dependent meiotic initiation and thus indicates separable functions for *IME4* in promoting meiosis and inhibiting PH development.

### Ime4 protein and mRNA methylation peak in pre-meiotic S and G2

**Figure 4:**
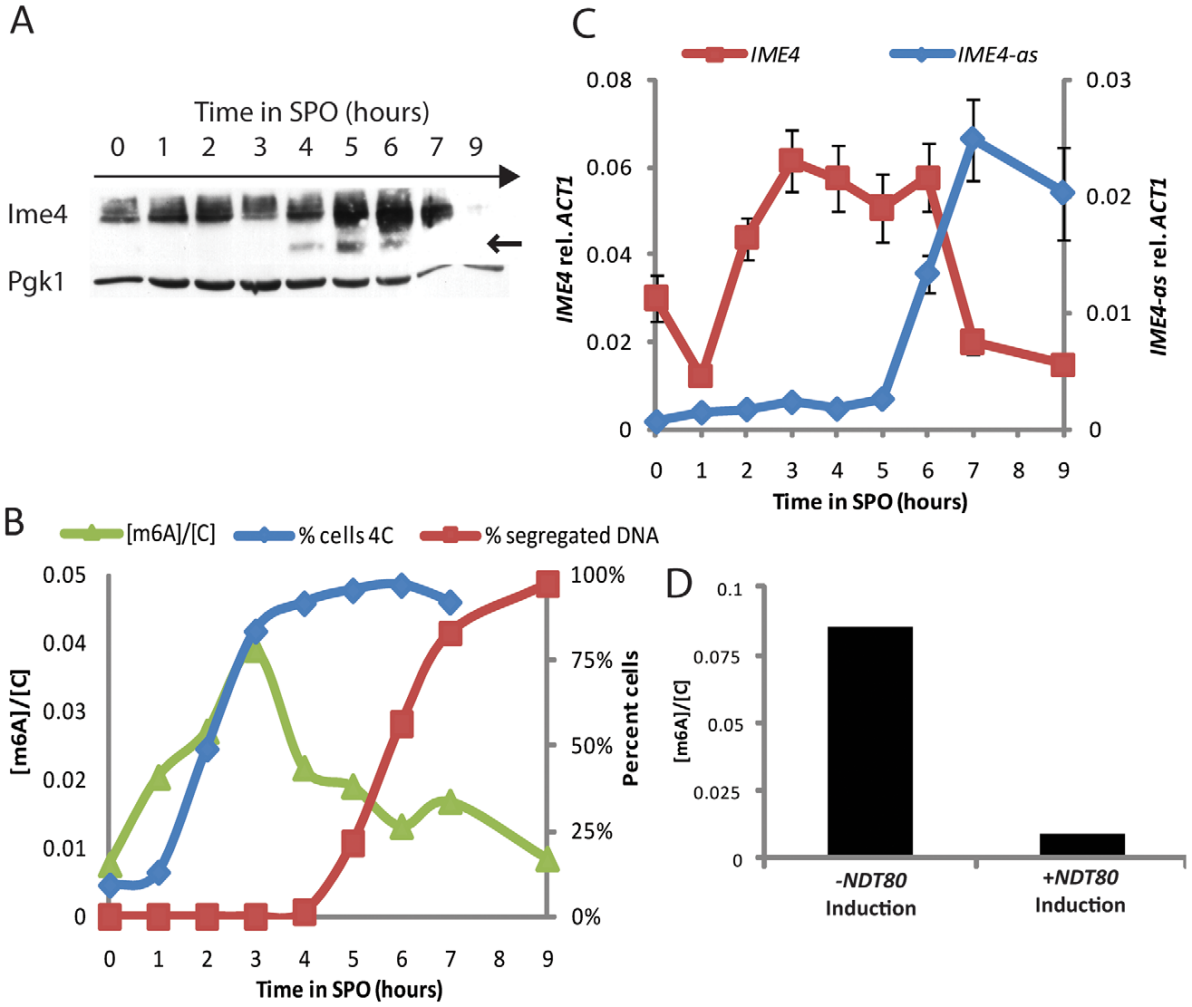
m^6^A accumulates prior to meiotic divisions. A) Western analysis for 3x-myc-tagged Ime4 protein (SAy914) throughout meiosis; Pgk1 protein serves as loading control. B) Quantification of m^6^A abundance relative to cytosine throughout meiosis (green triangles, left axis) in a wild-type strain (SAy821). Percent of 4C cells as quantified by FACS (3×10^4^ cells/time point—blue diamonds, right axis) and percent cells undergoing nuclear divisions as assayed by DAPI staining (200 cells/time point—red squares, right axis) are shown as references for meiotic progression. C) Strand-specific qPCR for sense *IME4* (red squares, left axis) and antisense transcript (*IME4-as*) (blue diamonds, right axis) transcript throughout meiosis. D) m^6^A relative to cytosine quantification in cells carrying an estradiol-inducible *NDT80* construct as their sole source of *NDT80* (SAy995). Cells were treated with β-estradiol or vehicle 6 hours after meiotic induction and monitored at 9 hours.

To determine the period during starvation when Ime4 is most abundant, we analyzed Ime4 protein levels by Western blotting in a synchronous time course after inoculation in SPO. As shown in Figure 4A, Ime4 levels increased soon after the shift to starvation conditions and peaked during pre-meiotic S and G2/prophase. Once cells entered into the meiotic divisions, full-length Ime4 disappeared rapidly. We also observed the accumulation of a faster-migrating band that may represent a carboxy-terminal cleavage product of Ime4 (Figure 4A).

To investigate whether Ime4 protein levels correlated with activity we followed the kinetics of m^6^A accumulation on polyadenylated RNA by two-dimensional thin-layer chromatography. This analysis revealed that m^6^A accumulation tightly matched the levels of full-length Ime4 during the starvation time course. m^6^A accumulated soon after the shift to SPO, peaked during pre-meiotic S and G2/prophase, and decreased as cells entered into the meiotic divisions (Figure 4B). Entry into the meiotic divisions resulted in a decrease in the *IME4* sense transcript and the concomitant accumulation of the *IME4* regulatory antisense transcript [10], [29] (Figure 4C), suggesting that *IME4* expression may be regulated both at the protein and transcriptional levels to ensure tight repression of *IME4*.

To determine more precisely the point at which m^6^A was lost we employed cells encoding an estradiol-inducible allele of *NDT80*, the transcription factor necessary for exit from meiotic G2/prophase [8], [30], [31]. In the absence of estradiol, these cells arrest at the end of G2/prophase [8], [32]. Under these conditions, cells continued to produce methylated transcripts even after 9 hours of starvation (Figure 4D). By contrast, cells that were induced to express *NDT80* displayed reduced levels of m^6^A at this time point. Thus, *NDT80* activation and exit from meiotic G2/prophase is necessary for the down-regulation of RNA methylation activity.

### Ime4 interacts with Mum2 and Slz1 to form a methyltransferase complex

**Figure 5:**
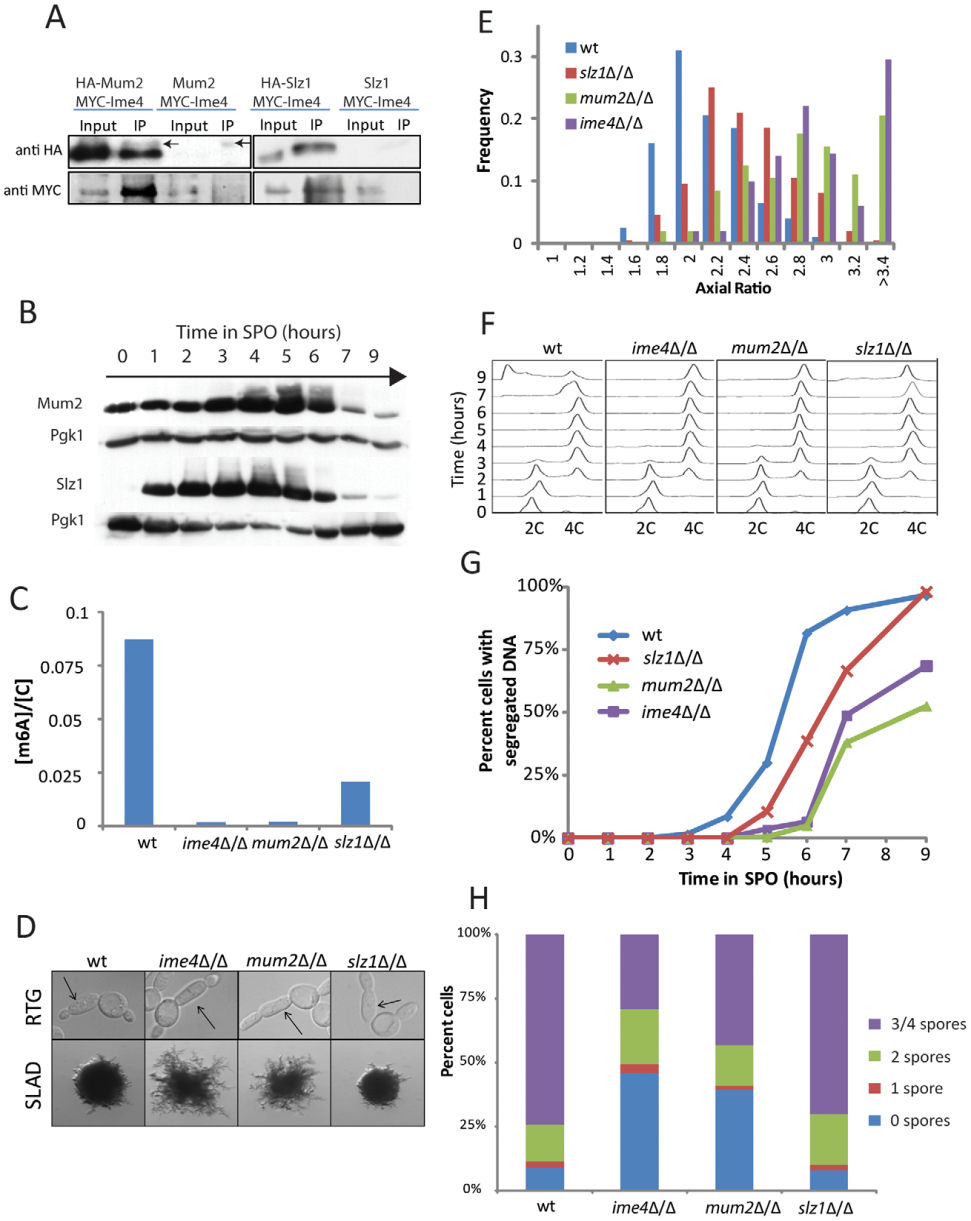
Mum2 and Slz1 interact with Ime4 and are required for m^6^A formation. A) Western analysis for co-immunoprecipitation of Mum2 (left panels) and Slz1 (right panels) with Ime4. HA-tagged Mum2 or Slz1 was immunoprecipitated from cellular extracts 3 hours after induction of meiosis and probed for interaction with myc-tagged Ime4 (SAy1232, SAy1253, respectively). A myc-Ime4 (SAy914) strain without HA-tags served as a control. Arrows in the IP lanes indicate IgG bands. B) Western analysis for HA-tagged Mum2 protein (SAy1235) or HA-tagged Slz1 protein (SAy1254) throughout meiosis; Pgk1 protein serves as loading control. C) Quantification of m^6^A on mRNA three hours after meiotic starvation, when m^6^A accumulation is maximal in wild-type cells (SAy821). Deleting any one of *ime4*Δ/Δ (SAy771), *mum2*Δ/Δ (SAy1196) and *slz1*Δ/Δ (SAy1206) results in a reduction in m^6^A levels. D) Wild-type (SAy821), *ime4*Δ/Δ (SAy771), *mum2*Δ/Δ (SAy1196) and *slz1*Δ/Δ (SAy1206) daughter cell morphology upon RTG_3_ (top panels). Arrows indicate primary buds. The same strains were photographed after growth on SLAD for 6 days (bottom panels). E) Axial ratio quantification of primary daughter cells upon RTG_3_ for strains in (D). (F) FACS analysis of DNA synthesis in strains from (D) throughout a meiotic time course (n = 3×10^4^ cells/strain/time point). DNA content of diploid cells before DNA replication (2C) and after DNA replication (4C) is indicated. G) Kinetics for meiotic nuclear divisions as assayed by DNA staining by DAPI in the strains from (C) (n = 200 cells/strain/time point). H) Number of asci with one, two, three/four, or no spores in strains from (D) after 24 hours in SPO medium (n = 200 cell/strain).

To identify regulators of Ime4, we conducted a two-hybrid screen using full-length Ime4 as bait [33]. The two most abundant hits isolated from this screen were *MUM2* (*MU*ddled *M*eiosis 2) and *SLZ1* (*S*porulation-specific *L*eucine *Z*ipper 1) both of which have previously been implicated in meiotic progression [34], [35], [36]. Our screen identified 28 independent clones of *MUM2* and 8 independent clones of *SLZ1*. All 28 clones spanned the 3′ region of *MUM2* and all 8 clones spanned the 3′ region of *SLZ1*, suggesting that the respective carboxy-terminal regions of these proteins are sufficient for conferring interaction with Ime4 (Figure S5). In support of the physical interactions revealed by two-hybrid analysis, Ime4 efficiently co-immunoprecipitated with Mum2 and, to a lesser extent, Slz1 (Figure 5A). Like Ime4, Mum2 and Slz1 were induced during starvation in SPO as determined by Western blotting (Figure 5B).

To test whether Mum2 and Slz1 acted together with Ime4 in controlling mRNA methylation during starvation, we quantified m^6^A levels in meiotic G2/prophase in cells lacking *IME4*, *MUM2* or *SLZ1*. As previously reported, *ime4*Δ/Δ cells did not accumulate m^6^A in meiosis [18]. Importantly, *mum2*Δ/Δ mutants also failed to accumulate m^6^A mRNA in pre-meiotic G2 and m^6^A in *slz1*Δ/Δ cells accumulated to substantially lower levels than in wild type cells (Figure 5C). Taken together, these findings indicate that Ime4, Mum2, and Slz1 bind to each other and function together to mediate m^6^A RNA methylation during starvation. We term this protein complex the MIS (Mum2, Ime4, Slz1) complex.

Consistent with their shared functions in mRNA methylation, *mum2*Δ/Δ and *slz1*Δ/Δ cells recapitulated the defects of *ime4*Δ/Δ mutants with respect to meiotic progression and PH development. Like *ime4*Δ/Δ, deletion of either *MUM2* or *SLZ1* resulted in hyper-filamentation upon RTG_3_ and on SLAD plates. The hyper-filamentation phenotype appeared less pronounced for *slz1*Δ/Δ cells (Figure 5D, 5E). Similarly, like *ime4*Δ/Δ cells, both *mum2*Δ/Δ and *slz1*Δ/Δ mutants replicated DNA under starvation conditions with essentially wild-type kinetics, but were delayed in progressing into the meiotic divisions (Figure 5F, 5G). Again, the delay was less severe for *slz1*Δ/Δ mutants than for *ime4*Δ/Δ or *mum2*Δ/Δ mutants, mirroring the less dramatic effect of *SLZ1* deletion on m^6^A levels. The loss of function mutations in *MUM2* and *SLZ1* had meiotic defects consistent with the other phenotypes: deletion of *MUM2* resulted in a spore formation defect similar to loss of *IME4*, whereas deletion of *SLZ1* did not appreciably affect spore formation (Figure 5H). We conclude that the MIS complex controls m^6^A RNA methylation and the yeast starvation response.

### Expression of the MIS complex is sufficient to confer mRNA methylation

**Figure 6:**
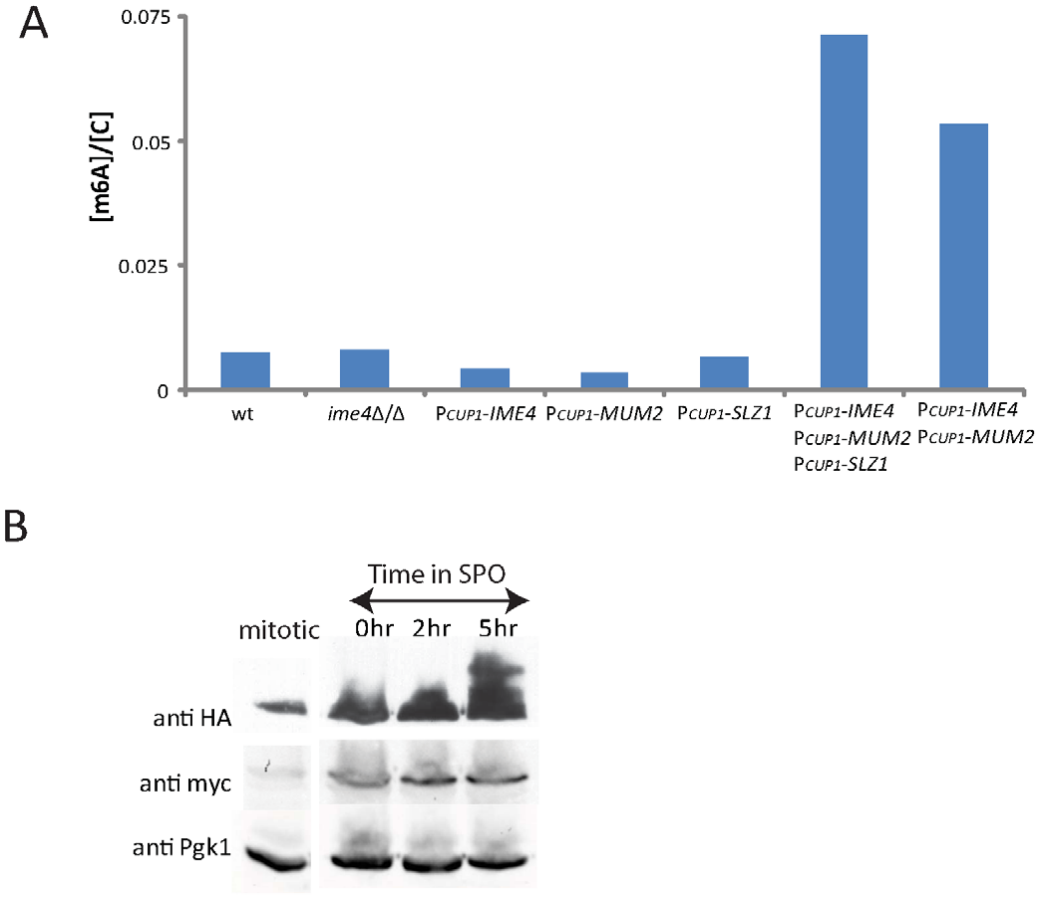
MIS complex expression is sufficient to induce m^6^A accumulation on mRNA. A) m^6^A accumulation on mRNA was quantified in rich conditions in wild-type (SAy821), *ime4*Δ/Δ (SAy771), P*_CUP1_*-*IME4* (SAy1249), P*_CUP1_*-*MUM2* (SAy1251), P*_CUP1_*-*SLZ1* (SAy1250), P*_CUP1_*-*IME4* P*_CUP1_*-*MUM2* P*_CUP1_*-*SLZ1* (SAy1248) and P*_CUP1_*-*IME4* P*_CUP1_*-*IME4* (SAy1252) after 150 minutes of mitotic growth in the presence of cupric sulfate. B) Western analysis for expression of Ime4 and Mum2. Cells encoding epitope-tagged Ime4 and Mum2 (SAy1232) were collected either from the rich cupric-sulfate media conditions in (A) (first column) or at 0, 2, and 5 hours in meiosis (as labeled), then subjected to Western analysis for either Ime4 (anti-myc) or Mum2 (anti-HA). Pgk1 serves as a loading control. Images across each row come from the same exposure.

The necessity of *IME4*, *MUM2*, and *SLZ1* for the methylation of mRNA during meiosis raised the question of whether expression of these genes was sufficient to induce the methylation of mRNA. To test this, one copy of each gene was placed under control of the inducible *CUP1* promoter in diploid cells while the other copy remained unaltered. Expression of these genes was induced by the addition of cupric sulfate in rich medium, a condition in which m^6^A does not normally accumulate on mRNA (Figure 6A). Under these growth conditions, neither Mum2 nor Ime4 were expressed at levels close to those found in meiosis (Figure 6B); Slz1 did not accumulate in cells until induction of meiotic development (Figure 5B). We found that inducing expression of *IME4*, *MUM2*, or *SLZ1* singly in these conditions was not sufficient to induce m^6^A accumulation on mRNA (Figure 6A). By contrast, induction of both *IME4* and *MUM2* resulted in a strong accumulation of m^6^A. Induction of all three MIS components (*MUM2*, *IME4*, and *SLZ1*) further elevated m^6^A, albeit only by a small fraction, consistent with the role of *SLZ1* as a non-essential component of the MIS complex (Figure 6A). Notably, none of the strains that express m^6^A in rich conditions exhibited any obvious morphological or growth differences as compared to un-induced control cells (data not shown). These data suggest that the restriction of mRNA methylation to times of starvation is largely a result of the starvation-specific expression of the MIS complex. Taken together, these phenotypes suggest a model in which Mum2 and Ime4 are essential components of an RNA methyltransferase complex, with Slz1 providing an accessory role necessary for optimal function.

### m^6^A down-regulation is necessary for RTG-PH development

**Figure 7:**
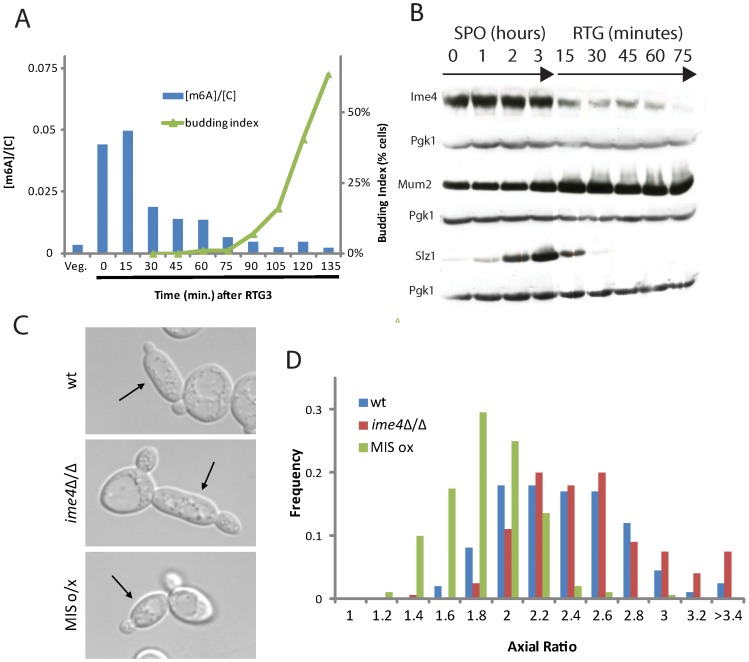
m^6^A formation inhibits filamentation. A) Quantification of m^6^A abundance relative to cytosine (blue bars, left axis) and budding index (green triangles, right axis) upon RTG_3_. B) Western analysis for 3x-myc-tagged Ime4 protein (SAy914), 3x-HA-tagged Mum2 protein (SAy1235) or 3x-HA-tagged Slz1 protein (SAy1254) throughout RTG_3_ (*i.e.*, following the shift to YPD after 3 hours in SPO); Pgk1 protein serves as loading control. C) Representative images of cells from wild-type (SAy821), *ime4*Δ/Δ (SAy771) and a strain induced to express the three components, *IME4*, *MUM2* and *SLZ1* (SAy1248) from P*_CUP1_* after RTG_3_. All strains were treated with cupric sulfate upon RTG_3_ into YPD. D) Axial ratio quantifications of RTG_3_ cells from cells in (C) (n = 200 cells/strain).

The MIS complex acts as an inhibitor of PH development, which raises the question of how cells are able to enter RTG-PH development during meiotic G2/prophase when the levels of MIS complex components and m^6^A are high. To answer this question, we monitored m^6^A levels upon RTG_3_. This analysis revealed that m^6^A levels dropped rapidly after RTG_3_, approaching the level of vegetative cells by 75 minutes after RTG_3_, shortly before bud formation was initiated in RTG cultures (Figure 7A). These observations suggest that MIS-dependent inhibition of PH development is alleviated in time to allow elongated bud growth in RTG_3_ cultures.

The drop in m^6^A levels upon RTG_3_ was accompanied by modification of MIS complex components. Western analysis revealed that Ime4 protein levels gradually decreased and were undetectable by 75 minutes after RTG_3_ (Figure 7B). Concomitantly, a fraction of Mum2 accumulated in a higher molecular-weight form, a modification that was also apparent when cells entered the meiotic divisions (Figure 5B). Similar to Ime4, Slz1 was quickly degraded and was no longer detectable by 30 minutes after RTG (Figure 7B). These data indicate that before initiating the PH developmental program, cells remove existing methylated RNA and, in parallel, deactivate the mRNA methyltransferase program by modifying or degrading components of the MIS complex before initiating the PH developmental program.

To test whether this down-regulation of the mRNA methyltransferase program is necessary for PH development upon RTG, we ectopically expressed the MIS complex components in RTG_3_ cells. As shown in Figure 7C and 7D, RTG_3_ cells expressing the MIS complex failed to form PH cells and instead developed ovoid buds. These data support the conclusion that mRNA methylation is inhibitory to PH development and suggest that the decrease in m^6^A expression upon RTG is necessary for PH cell development.

## Discussion

### Control of cell fate during starvation

Our results are consistent with a progressive restriction of diploid cell fate in response to severe nutrient deprivation. During the initial lineage restriction, which coincides with pre-meiotic DNA replication, cells commit to starvation-induced differentiation. In this state, cells remain bipotential, as they can either form spores or engage in PH development, depending on nutrient availability. Only after starvation conditions have persisted long enough to initiate the middle-meiotic program do cells become committed to meiosis and sporulation [22], [23]. The timing of the bipotential stage occurred after readiness-to-sporulate and before meiotic commitment, as defined by Simchen and colleagues [22].

We propose that the bipotential state is geared toward balancing the need to proliferate with survival under nutrient deprivation. Although the formation of protective spores provides better odds of survival for individual cells (or their meiotic offspring) in a harsh environment, sporulation is very time-intensive, which represents a competitive disadvantage if neighboring cells continue to proliferate. By maintaining the option to enter PH development while completing the early stages of sporulation, cells are primed to forage the environment and proliferate, should nutrient deprivation turn out to be transient.

### RTG as a tool for studying yeast development

The RTG regimen has proven a powerful tool for probing the yeast starvation response and has provided important insights into a variety of meiotic processes including meiotic commitment and DNA repair [4], [21], [22], [23], [37], [38]. Importantly, the RTG-PH procedure also offers a new avenue for dissecting the kinetics and biochemistry of PH development. Previous assays analyzed PH development in *S. cerevisiae* on solid medium or liquid suspension [3], [39], [40]. Under these conditions, PH cells form colonies comprised of a mixture of yeast form and PH cells that divide asynchronously with respect to each other and invade into agar. The asynchronous population of multiple cell types prohibited a biochemical analysis of gene expression in PH cells. Other studies have utilized butanol in liquid culture to study PH development [41], [42]. Although growth in butanol results in elongated cells, formation of those cells does not require *FLO11* or its regulators. By contrast, the genetic and morphological features of RTG-PH are indistinguishable from PH development on solid agar. The RTG-PH method thus provides an opportunity to study PH development in homogeneous and synchronous cultures.

### RNA methylation restricts cell fate

The RTG procedure enabled the discovery that a tightly controlled m^6^A mRNA methylation program governs cell fate restriction during starvation. m^6^A is an enigmatic mRNA modification that is highly increased during starvation in diploid yeast [11], [18] and may function to enhance translational activity [13], [16] or to regulate message processing and stability [14], [15]. Previous work had identified the meiotic inducer Ime4 as the enzyme necessary for m^6^A formation [18]. Our experiments show that Ime4 binds two additional proteins, Mum2 and Slz1, to form a protein complex, which we termed the MIS complex. All three proteins are required for efficient RNA methylation, indicating that Mum2 and Slz1 promote the methyltransferase activity of Ime4. There are no obvious protein domains that would indicate a specific function of either Mum2 or Slz1 other than the Slz1 leucine-zipper motif, a domain type that often functions in protein dimerization. However, based on the severity of the phenotypes associated with loss of these two proteins, we predict that Mum2 forms an integral activator of the MIS complex, possibly by activating Ime4 catalytic activity or by targeting Ime4 to mRNA substrates, whereas Slz1 likely only has accessory functions.

Our results suggest that accumulation of m^6^A mRNA is largely governed by regulating the abundance of MIS complex components. All three proteins are specifically expressed during pre-meiotic S and G2/prophase and are sufficient to induce m^6^A methylation in rich medium. Moreover, concomitant with the loss of m^6^A mRNA during the meiotic divisions or during return to growth, expression of MIS complex components drops and the proteins are rapidly modified or degraded. However, given the rapid drop in m^6^A mRNA observed after return to growth in particular, m^6^A mRNA may also become actively demethylated. In human cells, the protein FTO was recently shown to act as an mRNA demethylase [43], but homologues of FTO have thus far not been identified in yeast.

MIS-complex-dependent RNA methyltransferase activity governs multiple developmental processes during nutrient starvation. All three MIS complex components are required for efficient sporulation and may act at multiple steps during this process [18], [34], [36]. Ime4 and Mum2 had been shown to promote entry into the meiotic program and premeiotic DNA replication, respectively [18], [35], [36], although our results as well as previous observation suggest that the importance of these early roles varies with strain background [18]. Our findings indicate that RNA methylation also functions later in the meiotic program to promote the expression of the middle-meiosis transcription factor *NDT80* and thus meiotic commitment. Ime4 may activate *NDT80* expression either directly, by methylating the *NDT80* transcript, or indirectly, through the previously characterized modification of *IME2* transcript [17], as Ime2 activity is necessary for expression of Ndt80 protein [8]. Interestingly, *NDT80* is, in turn, required to down-regulate of mRNA methylation during the meiotic divisions revealing a negative feedback loop in switching off mRNA methylation.

MIS-complex activity suppresses PH development in a manner that is at least partially separable from its function in promoting meiosis and that involves down-regulation of the key PH factor, *FLO11*. Inhibition of *FLO11* expression may occur through methylation (and hence, activation) of *SFL1*, a well-characterized inhibitor of *FLO11* and the PH program [44]. Identification of specific substrates for mRNA methylation will be necessary to determine how Ime4 regulates these developmental decisions.

### Conservation of the MIS complex

Sequence and functional comparisons suggest that Ime4 and Mum2 are conserved throughout eukaryotic evolution. Previous biochemical studies in human cells isolated two independent components, with a possible third additional factor, as necessary for catalyzing mRNA methylation [45], [46]. Of these three components, only *MT-A70*, the human homolog of *IME4*, has been cloned [46]. This subunit is conserved throughout virtually all eukaryotes, including *Arabidopsis thaliana* and *Drosophila melanogaster* [12], [14], [46], [47]. Mum2 is similarly conserved; the *Arabidopsis MUM2* homolog, At*FIP37*, was previously found to interact with the *Arabidopsis IME4* homolog, *MTA*, although its role in RNA methylation was not determined [14]. *MUM2* also bears homology to *Drosophila melanogaster Fl(2)d* and human *WTAP-1*, the latter of which therefore is a strong candidate to be the cognate human mRNA methyltransferase component. Alignment of *MUM2*, *Fl(2)d*, *AtFIP37* and *WTAP-1* protein sequences revealed that *MUM2* is the most diverged from the other homologues, although all four genes have conserved residues near the C-terminus, a region we suggest is necessary for interaction with Ime4 (Figure S6). Intriguingly, *MUM2* and *AtFIP37* homologues in metazoans have been shown to play a role in mRNA splicing [48], [49], [50], [51]. Although the yeast genome is generally intron-poor, introns are strongly over-represented in early meiotic transcripts [52], [53], raising the possibility that mRNA methylation may influence the splicing of these transcripts to regulate the starvation-induced development in yeast. The homologues of *IME4* and *MUM2* are highly expressed in the reproductive organs as well as other developing tissues in higher eukaryotes [14], [47], [50], [54]. Thus, the control of developmental decisions by mRNA methylation may be widely conserved.

## Methods

### Strains and growth conditions

Strain genotypes are shown in Table S1. To induce synchronous meiotic entry, cells were pre-selected on 1% yeast extract, 2% peptone, 3% glycerol, 2% agar for 24 hours at 30°C, grown for 24 hr in 1% yeast extract, 2% peptone, 4% dextrose at 30°C, diluted in BYTA (1% yeast extract, 2% tryptone, 1% potassium acetate, 50 mM potassium phthalate) to OD_600_ = 0.2 and grown for another 16 hr at 30°C, 300 rpm. Cells were then washed once with water and re-suspended in SPO (0.3% potassium acetate) at OD_600_ = 2.0 and incubated at 30°C at 190 rpm. For RTG experiments, cells were removed from SPO at the indicated times, collected by centrifugation, re-suspended in pre-warmed 1% yeast extract, 2% peptone, 2% dextrose and incubated at 30°C at 190 rpm. Pseudohyphal growth was assayed after 6 days of growth on synthetic low-ammonium dextrose (SLAD) medium described in [2] containing 0.5% glucose. The *CUP1* promoter was induced with 100 µM CuSO_4_ in rich media. RTG cells were photographed and measured after one complete cell cycle, when the daughter cell initiated budding, in order to gauge maximal length of daughter buds. Two hybrid analysis was performed as in [33]. Full-length *IME4* was expressed as a fusion to the Gal4 DNA-binding domain and was transformed into a bait strain that was mated with the two-hybrid library. Plasmids from colonies that showed growth on auxotrophic media and expressed LacZ were further purified and sequenced.

### Cell morphology quantification

Cells were photographed under 40× magnification and primary bud morphology was quantified using ImageJ (Rasband W., National Institutes of Health, http://rsb.info.nih.gov/ij/index.html).

### Quantitative PCR

Total RNA was obtained by standard phenol∶chloroform∶isoamyl alcohol extraction. cDNA was generated using random hexamers or strand-specific primers and the Qiagen QuantiTect Reverse Transcription Kit. Transcript abundance was quantified using reagents from Applied Biosystems and the ABI 7500 real-time PCR system. Primer sequences are provided in Table S2.

### Co-immunoprecipitation

50 ml of meiotic culture was harvested 3 hours after meiotic induction in the presence of protease inhibitors (Complete protease inhibitors, Roche). Cells were washed once with 1M Tris-HCl, pH 7.5 and snap-frozen. Frozen pellets were resuspended in lysis buffer (150 mM NaCl, 50 mM Tris pH 7.5, 1% NP40, 10 mM PMSF, Complete mini protease inhibitors (Roche) at 2× concentration) and glass-bead homogenized three times for 5 minutes at 4°C. Debris was pelleted by centrifugation for 10 minutes, and supernatant was incubated with HA-conjugated agarose beads (Pierce) with head-over-tail rotation for three hours. Beads were washed 5times in lysis buffer and boiled in reducing loading buffer, followed by the standard protocol for Western analysis as described below.

### Other techniques

Flow cytometric analysis of DNA content, 4′,6-diamidino-2-phenylindole (DAPI) staining for DNA segregation analysis and cell staging by spindle morphology using tubulin indirect immunofluorescence were performed as described in [55]. Western analyses were performed as described in [56], with anti-c-myc (9E10, Covance) or anti-HA (HA.11, Covance) at a concentration of 1∶1000. TLC analysis was carried out as in [14]. RNA was extracted from cells using a standard hot acid phenol protocol; poly(A) RNA was purified from total RNA with the Dynabeads mRNA purification system (Invitrogen) and analyzed on cellulose plates (20 cm×20 cm) from EMD.

## Supporting Information

Figure S1 Description of the cell cycle of RTG_3_ cells. A) Comparison of cell cycles of RTG_0_ (top panel) and RTG_3_ cells (bottom panel) using budding index (n = 200 cells/time point, blue diamonds), DNA content (n = 3×10^4^ cells/time point, red squares), percent metaphase spindles (green triangles) and % anaphase spindles (purple crosses) (n = 200 cells/time point) in wild-type cells (SAy821). Dashed vertical line in bottom graph represents time of shift to rich medium (YPD) from meiosis-inducing medium (SPO) for RTG_3_ cells. B) Measurement of nascent bud length in wild-type (SAy821) mother (x-axis) and daughter cells (y-axis) for RTG_0_ (red diamonds) and RTG_3_ (blue squares) (n = 50 cells/condition). Here, bud length quantifies time of budding: early-initiated buds will have a greater length as compared to buds that are initiated later. Like PH cells, RTG_3_ cells initiate budding synchronously between mother and daughter cells. Extrapolating back to mother bud length = 0 in the RTG_3_ situation, cells have a small, positive y-intercept, indicative of synchronous, if not precocious, daughter cell bud initiation prior to mother bud initiation, as previously reported in PH cells [3]. In contrast, extrapolating back to daughter bud length = 0, we found that RTG_0_ cells have a positive x-intercept, indicative of mother cell bud initiation prior to the onset of daughter bud formation, as in vegetative growth. Thus, whereas mother cells initiate budding prior to bud initiation in the daughter cell in vegetative cells, both mother and daughter cells initiate budding synchronously in the PH cell cycle. Plotting the mother bud length versus daughter bud length for both RTG_0_ and RTG_3_ conditions, we found that in both cases the slope of the regression comparing the daughter and mother bud lengths were comparable, suggesting that bud growth rate between mother and daughter cells are comparable between the RTG_0_ and RTG_3_ cases.
(TIF)

Figure S2 Pre-meiotic DNA synthesis is not necessary for RTG_3_ PH cell formation. A) DNA synthesis profiles of wild-type cells (SAy821) (left panel) or wild-type cells treated with 20 mM hydroxyurea (right panel) in meiosis (n = 3×10^4^ cells/time point). B) Cells from (A) were returned to growth at three hours after meiotic initiation and were allowed to develop initial buds. Axial ratios (n = 200 cells/condition) are quantified in (C). D) DNA synthesis of wild-type (SAy821) (left panel) and *clb5*Δ/Δ *clb6*Δ/Δ (SAy1087) cells (right panel) (n = 3×10^4^ cells/strain/time point). RTG_0_ (blue bars) and RTG_3_ (red bars) axial ratios were quantified in (E) (n = 200 cells/strain). F) Representative colony morphology of strains in (D) after developing 6 days on SLAD medium. G) DNA synthesis profiles of wild-type treated with HU in SPO (SAy821) (top) or *clb5*Δ/Δ *clb6*Δ/Δ (SAy1087) (bottom) cells after between washed and returned to growth at 3 hours after meiotic induction into rich medium without HU. DNA content is shown in blue diamonds, while budding index is represented in red boxes. Cells were shifted into rich medium from SPO after 180 minutes, as indicated with a vertical dashed line. H) “Readiness" assayed in cells progressing through meiosis. Cells were either removed from SPO after 0, 1, 2, 3 hours, washed and shifted into water, or maintained in SPO (as labeled). Percentage of cells that formed spores after 24 hours in water are quantified in blue bars (left axis). Red squares represent the percent of cells that were 4C at the time of shift from SPO to water (right axis) (n = 3×10^4^ cells/time point).
(TIF)

Figure S3 RTG of *ime2*Δ/Δ mutants after DNA replication results in PH cell development. A) Representative images of cells from *ime2*Δ/Δ (SAy859), and *ime2*Δ/Δ *ime4*Δ/Δ (SAy1123) after RTG_6_. Arrows indicate primary buds. B) Quantification of axial ratios of RTG_3_ cells from (A) (n = 200 cells/strain).
(TIF)

Figure S4 *FLO* genes are required for *ime4Δ/Δ* hyper-PH development. A) Representative images of cells from wild-type (SAy821), *ime4*Δ/Δ (SAy771), *ime4*Δ/Δ *flo11*Δ/Δ (SAy890) and *ime4*Δ/Δ *flo8*Δ/Δ (SAy938) after RTG_3_. Arrows indicate primary buds. B) Quantification of axial ratios of RTG_3_ cells from (A) (n = 200 cells/strain). C) Representative images from colonies from (A) grown on SLAD for 6 days.
(TIF)

Figure S5 Ime4-interacting clones of Mum2 and Slz1. Map of clones of *MUM2* (A) and *SLZ1* (B) isolated from a yeast two-hybrid screen. X-axis represents positions on chromosome II or XIV, respectively. Independent clones are represented above the x-axis. Putative Ime4-interaction domains with Mum2 and Slz1 as defined from the clones are highlighted in blue.
(TIF)

Figure S6 Conservation of Mum2. A) Dendrogram for Mum2 homologues, FL(2)D—*Drosophila melanogaster*, WTAP-1—*Homo sapiens*, *AtFIP37*—*Arabidopsis thaliana* and yeast Mum2, which serves as an outgroup. B) Alignment of protein sequences in (A). Blue squares represent partial homology, yellow squares represent partial identity, green squares (also starred) represent conserved identity.
(TIF)

Table S1 Strains and genotypes. All strains are of the SK1 background.
(DOCX)

Table S2 Primer sequences used in this study.
(DOCX)
