# Supplementary figures and images for: RNA Methylation by the MIS Complex Regulates a Cell Fate Decision in Yeast
Source: PLoS Genet. 2012 Jun 7;8(6):e1002732. doi: 10.1371/journal.pgen.1002732 (PMC3369947; doi:10.1371/journal.pgen.1002732)

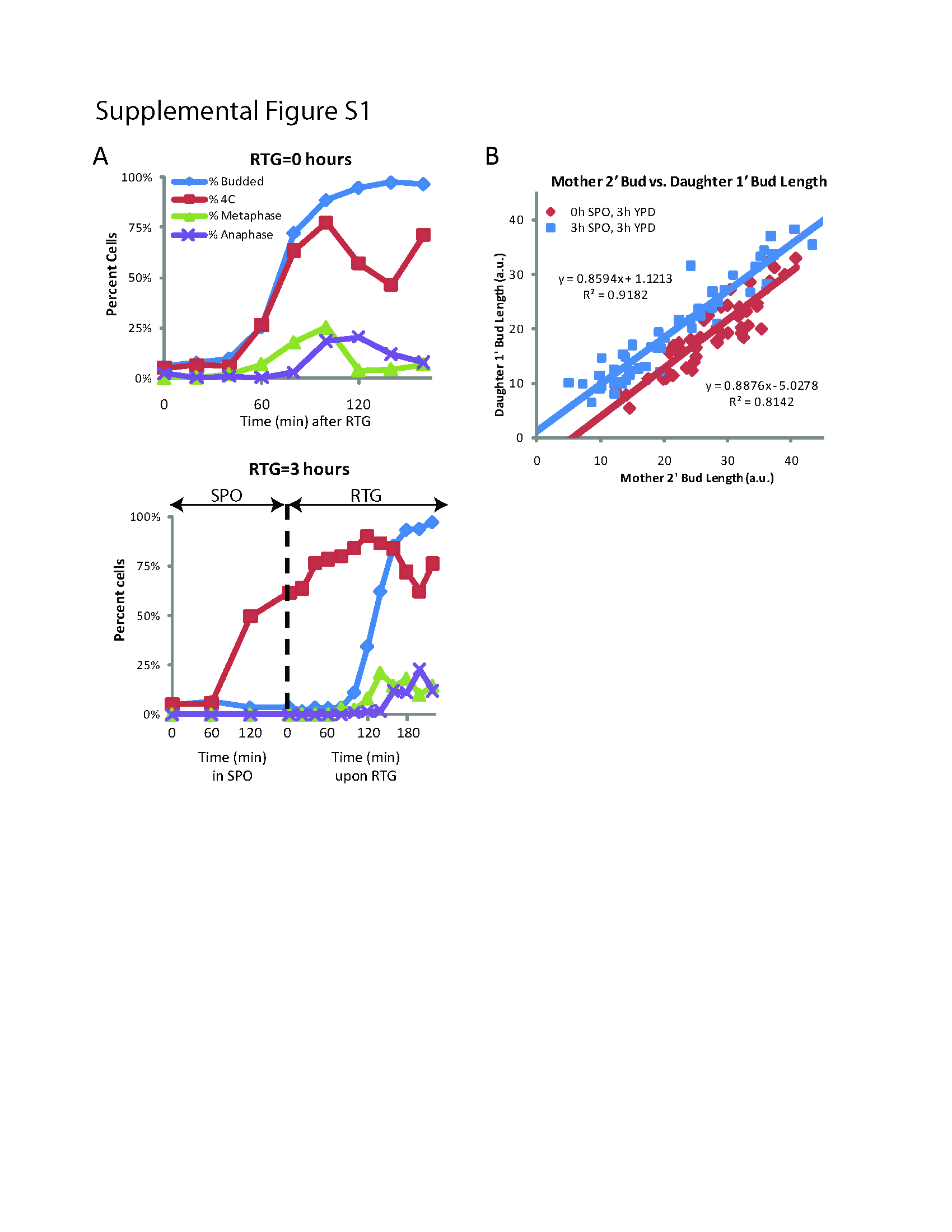

Supplement: Figure S1 — Description of the cell cycle of RTG3 cells. A) Comparison of cell cycles of RTG0 (top panel) and RTG3 cells (bottom panel) using budding index (n = 200 cells/time point, blue diamonds), DNA content (n = 3×10^4^ cells/time point, red squares), percent metaphase spindles (green triangles) and % anaphase spindles (purple crosses) (n = 200 cells/time point) in wild-type cells (SAy821). Dashed vertical line in bottom graph represents time of shift to rich medium (YPD) from meiosis-inducing medium (SPO) for RTG3 cells. B) Measurement of nascent bud length in wild-type (SAy821) mother (x-axis) and daughter cells (y-axis) for RTG0 (red diamonds) and RTG3 (blue squares) (n = 50 cells/condition). Here, bud length quantifies time of budding: early-initiated buds will have a greater length as compared to buds that are initiated later. Like PH cells, RTG3 cells initiate budding synchronously between mother and daughter cells. Extrapolating back to mother bud length = 0 in the RTG3 situation, cells have a small, positive y-intercept, indicative of synchronous, if not precocious, daughter cell bud initiation prior to mother bud initiation, as previously reported in PH cells [3]. In contrast, extrapolating back to daughter bud length = 0, we found that RTG0 cells have a positive x-intercept, indicative of mother cell bud initiation prior to the onset of daughter bud formation, as in vegetative growth. Thus, whereas mother cells initiate budding prior to bud initiation in the daughter cell in vegetative cells, both mother and daughter cells initiate budding synchronously in the PH cell cycle. Plotting the mother bud length versus daughter bud length for both RTG0 and RTG3 conditions, we found that in both cases the slope of the regression comparing the daughter and mother bud lengths were comparable, suggesting that bud growth rate between mother and daughter cells are comparable between the RTG0 and RTG3 cases. (TIF) [file pgen.1002732.s001.tif]

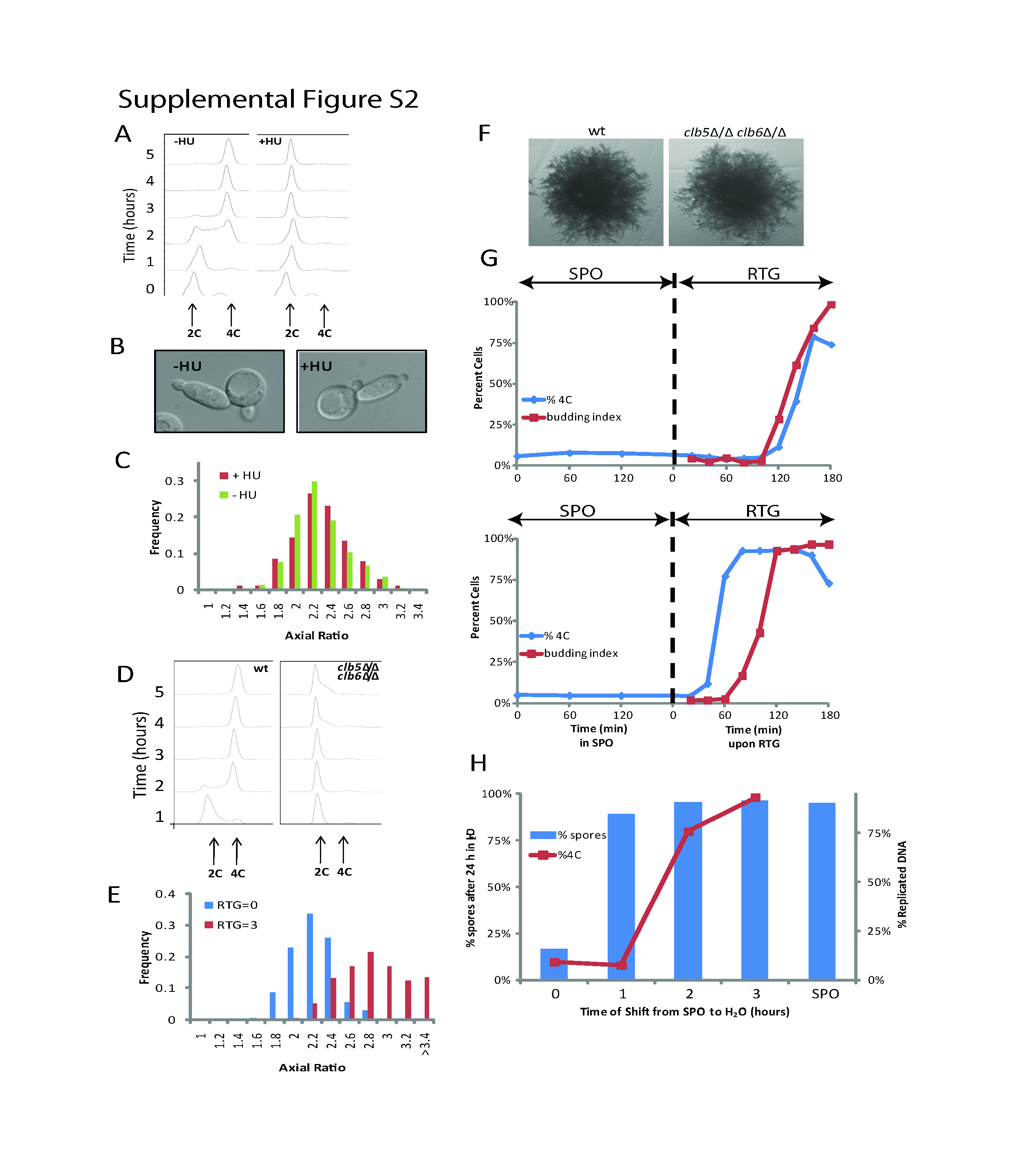

Supplement: Figure S2 — Pre-meiotic DNA synthesis is not necessary for RTG3 PH cell formation. A) DNA synthesis profiles of wild-type cells (SAy821) (left panel) or wild-type cells treated with 20 mM hydroxyurea (right panel) in meiosis (n = 3×10^4^ cells/time point). B) Cells from (A) were returned to growth at three hours after meiotic initiation and were allowed to develop initial buds. Axial ratios (n = 200 cells/condition) are quantified in (C). D) DNA synthesis of wild-type (SAy821) (left panel) and clb5Δ/Δ clb6Δ/Δ (SAy1087) cells (right panel) (n = 3×10^4^ cells/strain/time point). RTG0 (blue bars) and RTG3 (red bars) axial ratios were quantified in (E) (n = 200 cells/strain). F) Representative colony morphology of strains in (D) after developing 6 days on SLAD medium. G) DNA synthesis profiles of wild-type treated with HU in SPO (SAy821) (top) or clb5Δ/Δ clb6Δ/Δ (SAy1087) (bottom) cells after between washed and returned to growth at 3 hours after meiotic induction into rich medium without HU. DNA content is shown in blue diamonds, while budding index is represented in red boxes. Cells were shifted into rich medium from SPO after 180 minutes, as indicated with a vertical dashed line. H) “Readiness" assayed in cells progressing through meiosis. Cells were either removed from SPO after 0, 1, 2, 3 hours, washed and shifted into water, or maintained in SPO (as labeled). Percentage of cells that formed spores after 24 hours in water are quantified in blue bars (left axis). Red squares represent the percent of cells that were 4C at the time of shift from SPO to water (right axis) (n = 3×10^4^ cells/time point). (TIF) [file pgen.1002732.s002.tif]

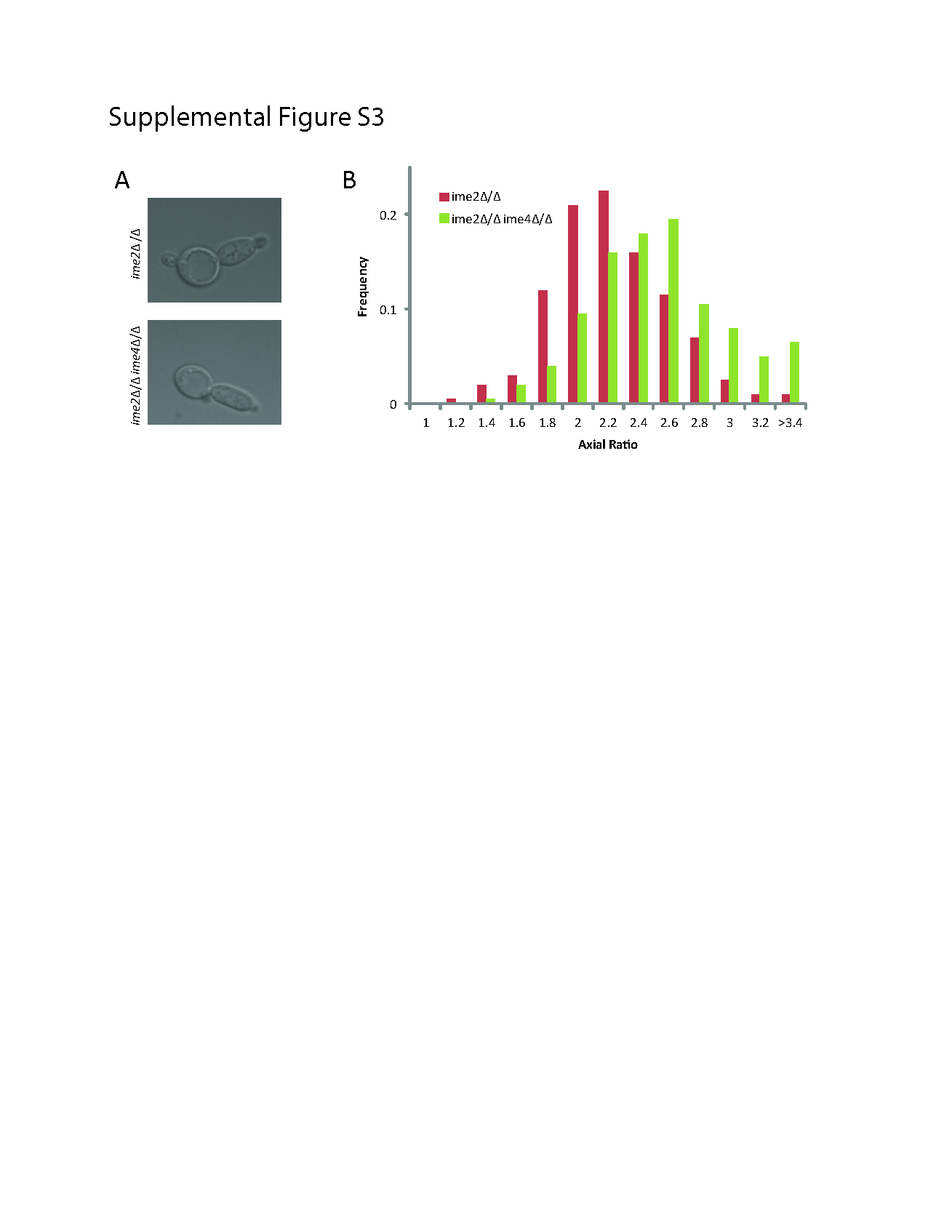

Supplement: Figure S3 — RTG of ime2Δ/Δ mutants after DNA replication results in PH cell development. A) Representative images of cells from ime2Δ/Δ (SAy859), and ime2Δ/Δ ime4Δ/Δ (SAy1123) after RTG6. Arrows indicate primary buds. B) Quantification of axial ratios of RTG3 cells from (A) (n = 200 cells/strain). (TIF) [file pgen.1002732.s003.tif]

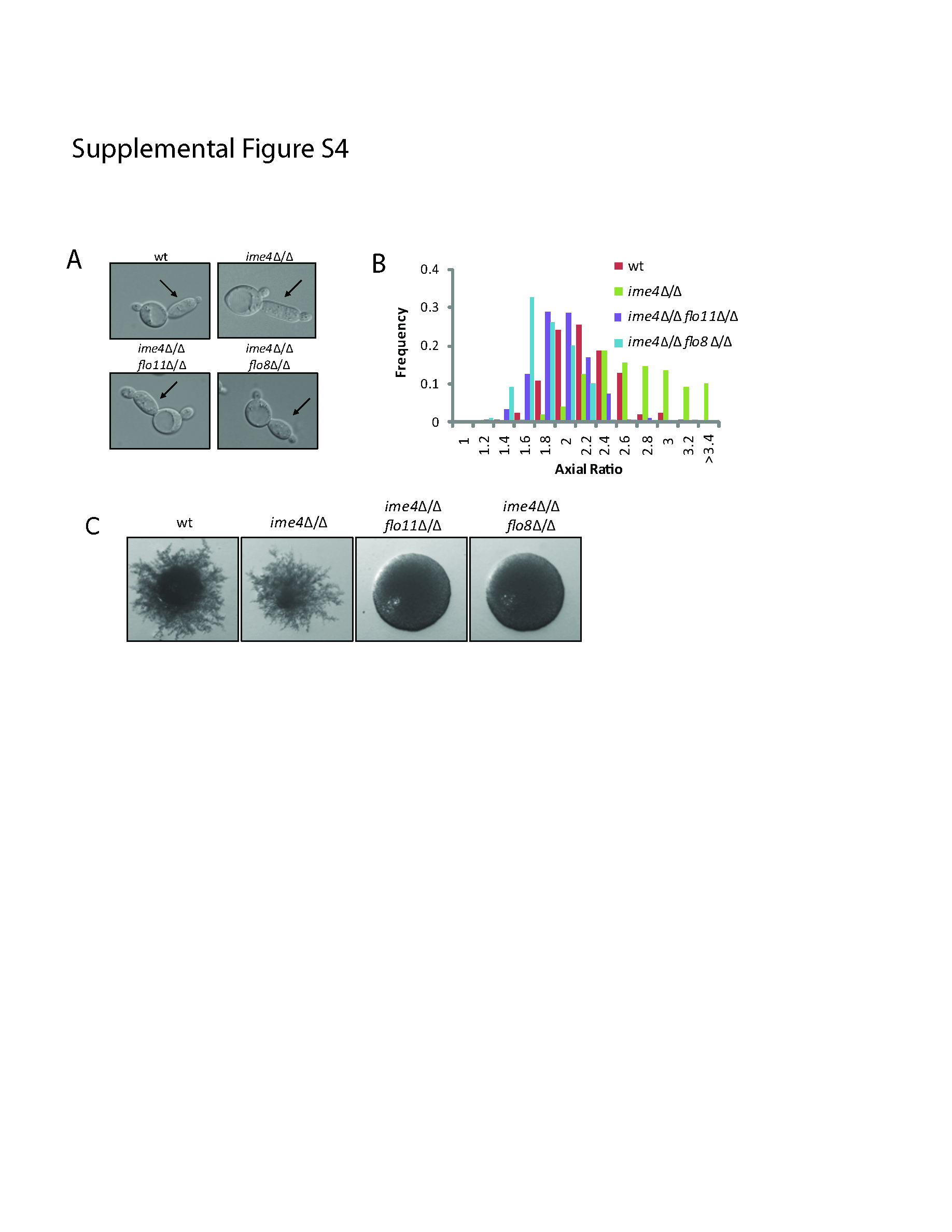

Supplement: Figure S4 — FLO genes are required for ime4Δ/Δ hyper-PH development. A) Representative images of cells from wild-type (SAy821), ime4Δ/Δ (SAy771), ime4Δ/Δ flo11Δ/Δ (SAy890) and ime4Δ/Δ flo8Δ/Δ (SAy938) after RTG3. Arrows indicate primary buds. B) Quantification of axial ratios of RTG3 cells from (A) (n = 200 cells/strain). C) Representative images from colonies from (A) grown on SLAD for 6 days. (TIF) [file pgen.1002732.s004.tif]

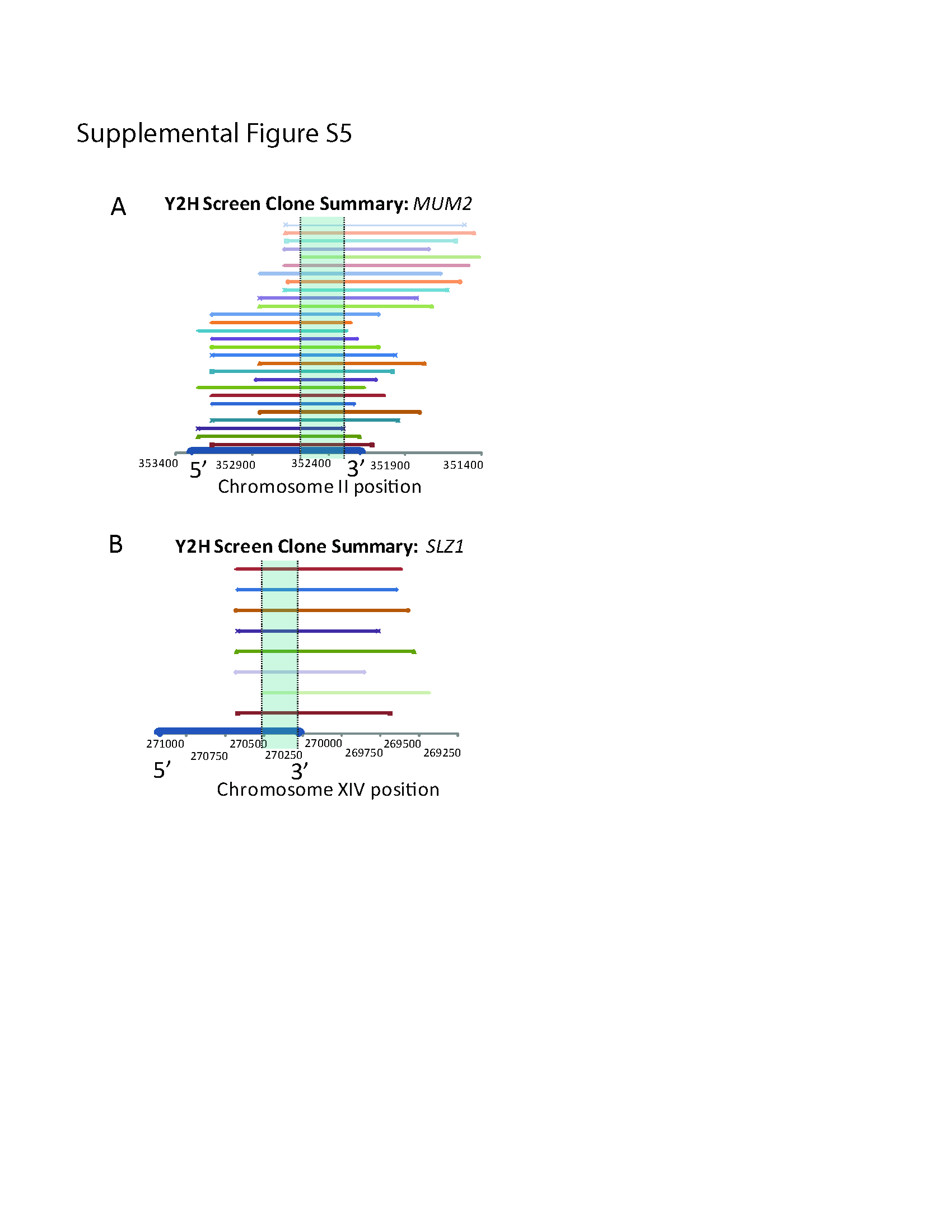

Supplement: Figure S5 — Ime4-interacting clones of Mum2 and Slz1. Map of clones of MUM2 (A) and SLZ1 (B) isolated from a yeast two-hybrid screen. X-axis represents positions on chromosome II or XIV, respectively. Independent clones are represented above the x-axis. Putative Ime4-interaction domains with Mum2 and Slz1 as defined from the clones are highlighted in blue. (TIF) [file pgen.1002732.s005.tif]

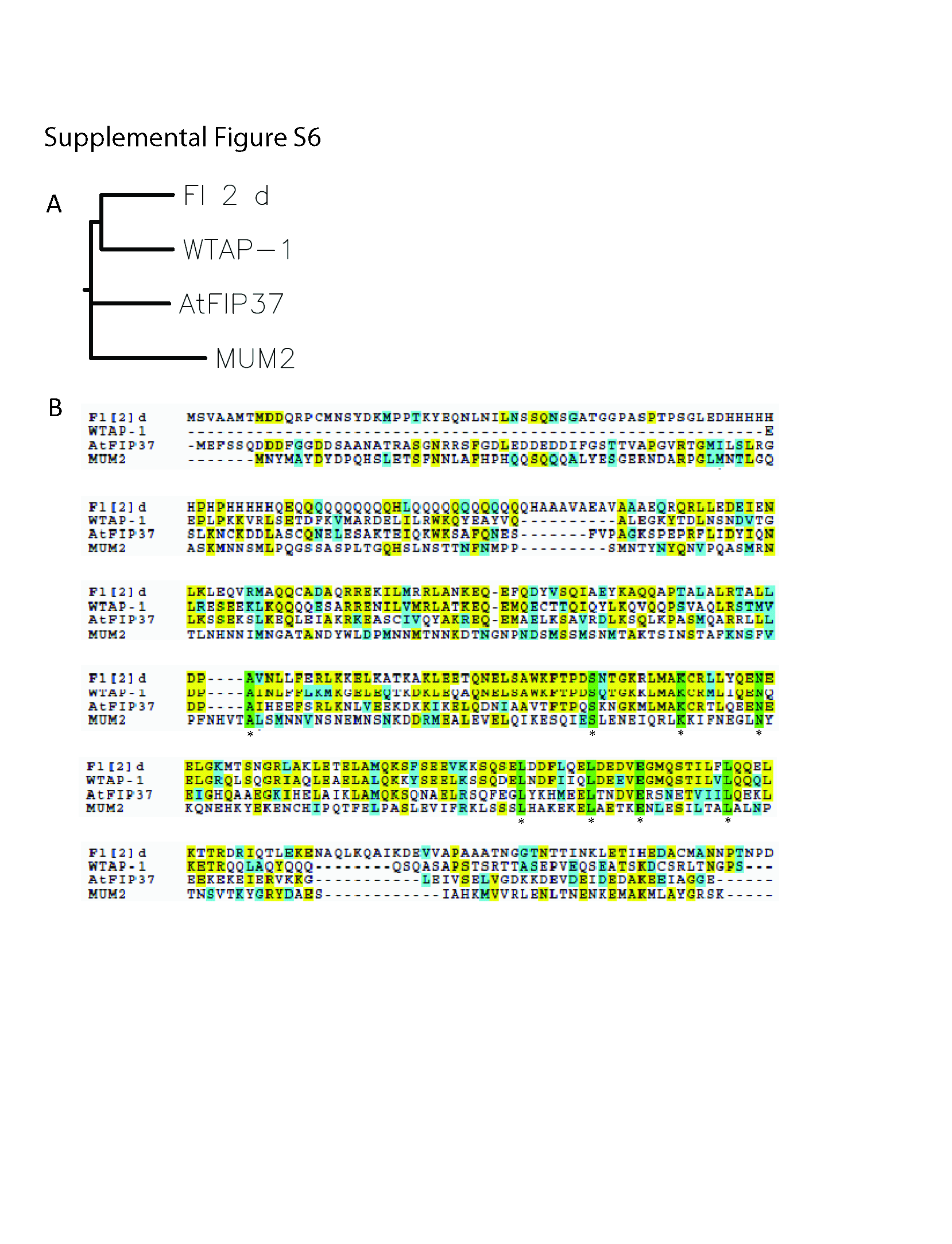

Supplement: Figure S6 — Conservation of Mum2. A) Dendrogram for Mum2 homologues, FL(2)D—Drosophila melanogaster, WTAP-1—Homo sapiens, AtFIP37—Arabidopsis thaliana and yeast Mum2, which serves as an outgroup. B) Alignment of protein sequences in (A). Blue squares represent partial homology, yellow squares represent partial identity, green squares (also starred) represent conserved identity. (TIF) [file pgen.1002732.s006.tif]
