# Supplementary material for: RNA Methylation by the MIS Complex Regulates a Cell Fate Decision in Yeast
Source: PLoS Genet. 2012 Jun 7;8(6):e1002732. doi: 10.1371/journal.pgen.1002732 (PMC3369947; doi:10.1371/journal.pgen.1002732)
Supplement: Table S1 — Strains and genotypes. All strains are of the SK1 background. (DOCX) [file pgen.1002732.s007.docx]

**Supplemental Table 1.** Strains and genotypes. All strains are of the SK1 background.

| **Strain #** | **Genotype** | **Source** |
| --- | --- | --- |
| **H224** | *MAT* α *lys2* *ho*::*LYS2* | Hochwagen |
| **SAy771** | *MAT* **a**/α *lys2*/*lys2* *ho*::*LYS2*/*ho*::*LYS2 LEU2*/*leu2 ime4*::*HIS3*/*ime4*::*HIS3* | This study |
| **SAy789** | *MAT* **a**/α *lys2*/*lys2* *ho*::*LYS2*/*ho*::*LYS2* *flo11*::KanMX4/*flo11*::KanMX4 | This study |
| **SAy821** | *MAT* **a**/α *lys2*/*lys2* *ho*::*LYS2*/*ho*::*LYS2* | This study |
| **SAy834** | *MAT* **a**/α *lys2*/*lys2* *ho*::*LYS2*/*ho*::*LYS2 ime1*::HIS3MX6/*ime1*::HIS3mx6 | This study |
| **SAy859** | *MAT* **a**/α *lys2*/*lys2* *ho*::*LYS2*/*ho*::*LYS2 ime2*::KanMX4/*ime2*::KanMX4 | This study |
| **SAy890** | *MAT* **a**/α *lys2*/*lys2* *ho*::*LYS2*/*ho*::*LYS2 LEU2*/*leu2 ime4*::*HIS3*/*ime4*::*HIS3* *flo11*::KanMX4/*flo11*::KanMX4 | This study |
| **SAy905** | *MAT* **a**/α *lys2*/*lys2* *ho*::*LYS2*/*ho*::*LYS2 flo8*::KanMX4/*flo8*::KanMX4 | This study |
| **SAy914** | *MAT* **a**/α *lys2*/*lys2* *ho*::*LYS2*/*ho*::*LYS2* 3xmyc-*IME4*/3xmyc-*IME4* | This study |
| **SAy938** | *MAT* **a**/α *lys2*/*lys2* *ho*::*LYS2*/*ho*::*LYS2 LEU2*/*leu2 ime4*::*HIS3*/*ime4*::*HIS3 flo8*::KanMX4/*flo8*::KanMX4 | This study |
| **SAy995** | *MAT* **a**/alpha *lys2*/*lys2* *ho*::*LYS2*/*ho*::*LYS2 leu2*::hisG/*LEU2* *his3*::hisG/*HIS3* *trp1*::hisG/*TRP1* P*_GAL1_*-*NDT80*::*TRP1*/*ndt80*::*LEU2*, *ura3*:::P*_GPD1_*-*GAL4*(848).ER::*URA3*/*URA3* | This study |
| **SAy1086** | *MAT* **a**/α *lys2*/*lys2* *ho*::*LYS2*/*ho*::*LYS2 ime4-D349A,W351A/ime4-D349A,W351A* | This study |
| **SAy1087** | *MAT* **a**/α *lys2*/*lys2* *ho*::*LYS2*/*ho*::*LYS2 HIS3*/*his3* *clb5*::*TRP1*/*clb5*::*TRP1* *clb6*::KanMX4/*clb6*::KanMX4 | This study |
| **SAy1123** | *MAT* **a**/α *lys2*/*lys2* *ho*::*LYS2*/*ho*::*LYS2 LEU2*/*leu2 ime4*::*HIS3*/*ime4*::*HIS3* *ime2*::KanMX4/*ime2*::KanMX4 | This study |
| **SAy1196** | *MAT* **a**/α *lys2*/*lys2* *ho*::*LYS2*/*ho*::*LYS2 mum2*::*HIS3*/*mum2*::*HIS3* | This study |
| **SAy1206** | *MAT* **a**/α *lys2*/*lys2* *ho*::*LYS2*/*ho*::*LYS2 slz1*::KanMX4/*slz1*::KanMX4 | This study |
| **A14154** | *MAT* **a**, *ho*::*LYS2*, *lys2*, *ura3*, *leu2*::hisG, *his3*::hisG, *trp1*::hisG, P*_GAL1_*-*NDT80*::*TRP1*, *ura3*:::P_GPD1_-*GAL4*(848).ER::*URA3* | [[6](#_ENREF_6),[27](#_ENREF_27)] |
| **SAy1232** | *MAT* **a**/α *lys2*/*lys2* *ho*::*LYS2*/*ho*::*LYS2 LEU2*/*leu2* *TRP1*/*trp1* 3xHA-*MUM2*/3xHA-*MUM2* 3xmyc-*IME4*/3xmyc-*IME4* | This study |
| **SAy1235** | *MAT* **a**/α *lys2*/*lys2* *ho*::*LYS2*/*ho*::*LYS2 LEU2*/*leu2* *TRP1*/*trp1* 3xHA-*MUM2*/3xHA-*MUM2* | This study |
| **SAy1248** | *MAT* **a**/alpha *lys2*/*lys2* *ho*::*LYS2*/*ho*::*LYS2 ime4*::P*_CUP1_*-*IME4*::KanMX4/*IME4* *mum2*::P*_CUP1_*-*MUM2*::KanMX4/*MUM2* *slz1*::P*_CUP1_*-*SLZ1*::KanMX4/*SLZ1* | This study |
| **SAy1249** | *MAT* **a**/alpha *lys2*/*lys2* *ho*::*LYS2*/*ho*::*LYS2 ime4*::P*_CUP1_*-*IME4*::KanMX4/*IME4* | This study |
| **SAy1250** | *MAT* **a**/alpha *lys2*/*lys2* *ho*::*LYS2*/*ho*::*LYS2* *slz1*::P*_CUP1_*-*SLZ1*::KanMX4/*SLZ1* | This study |
| **SAy1251** | *MAT* **a**/alpha *lys2*/*lys2* *ho*::*LYS2*/*ho*::*LYS2 mum2*::P*_CUP1_*-*MUM2*::KanMX4/*MUM2* | This study |
| **SAy1252** | *MAT* **a**/alpha *lys2*/*lys2* *ho*::*LYS2*/*ho*::*LYS2 ime4*::P*_CUP1_*-*IME4*::KanMX4/*IME4* *mum2*::P*_CUP1_*-*MUM2*::KanMX4/*MUM2* | This study |
| **SAy1253** | *MAT* **a**/α *lys2*/*lys2* *ho*::*LYS2*/*ho*::*LYS2 LEU2*/*leu2* *TRP1*/*trp1* 3xHA-*SLZ1*/3xHA-*SLZ1* 3xmyc-*IME4*/3xmyc-*IME4* | This study |
| **SAy1254** | *MAT* **a**/α *lys2*/*lys2* *ho*::*LYS2*/*ho*::*LYS2 LEU2*/*leu2* *TRP1*/*trp1* 3xHA-*SLZ1*/3xHA-*SLZ1* | This study |
