# Supplementary material for: RNA Methylation by the MIS Complex Regulates a Cell Fate Decision in Yeast
Source: PLoS Genet. 2012 Jun 7;8(6):e1002732. doi: 10.1371/journal.pgen.1002732 (PMC3369947; doi:10.1371/journal.pgen.1002732)
Supplement: Table S2 — Primer sequences used in this study. (DOCX) [file pgen.1002732.s008.docx]

**Supplemental Table 2.** Primer sequences:

| **Gene Name/Purpose:** | Forward primer | Reverse Primer |
| --- | --- | --- |
| *IME4* sense reverse transcription cDNA primer | ttttttagtaccagtgtggctgg |  |
| *IME4* antisense (*IME4-as*) reverse transcription cDNA primer | aatgaccagagagactagtagaaagc |  |
| *IME4* qPCR | gcggcctggctggttt | ccatttcgtaaatgcaatttcct |
| *NDT80* qPCR | ggcctccatctcgcctatc | cggatcctcattttccttcga |
| *ACT1* sense reverse transcription cDNA primer | tgaccatctggaagttcgtaggatt |  |
| *ACT1* qPCR | ctccaccactgctgaaagagaa | ccaaggcgacgtaacatagtttt |
